# The Efficacy and Pharmacological Mechanism of Zn_7_MT3 to Protect against Alzheimer’s Disease

**DOI:** 10.1038/s41598-017-12800-x

**Published:** 2017-10-23

**Authors:** Wei Xu, Qiming Xu, Hao Cheng, Xiangshi Tan

**Affiliations:** 1 Department of Chemistry & Institutes of Biomedical Sciences, Fudan University, Shanghai, 200433 China; 2 College of Biological & Chemical Sciences and Engineering, Jiaxing University, Jiaxing, 314001 China

## Abstract

Alzheimer’s disease (AD) is one of the leading causes of death for people over 65 years. Worse still, no completely effective therapeutic agent is available so far. One important pathological hallmark of AD is accumulated amyloid-β (Aβ) plaques with dysregulated metal homeostasis. Human metallothionin 3 (MT3), a regulator of metal homeostasis, is downregulated at least 30% in AD brain. So far, some *in vitro* studies demonstrated its multiple functions related to AD. However, it is a great pity that systematic *in vivo* studies of MT3 on AD model animals are still a blank so far. In this study, we treated APP/PS1 mice with sustained drug release of Zn_7_MT3 directly to the central nervous system, and investigated the role and molecular mechanism of Zn_7_MT3 to protect against AD mice systematically. The results demonstrated that Zn_7_MT3 can significantly ameliorate cognitive deficits, regulate metal homeostasis, abolish Aβ plaque load, and reduce oxidative stress. Additionally, it has been confirmed that MT3 is penetrable to the blood brain barrier of AD mice. All these results support that Zn_7_MT3 is an effective AD suppressing agent and has potential for applications in Alzheimer’s disease therapy.

## Introduction

Alzheimer’s disease (AD) is the most frequent form of neurodegenerative disease, which accounts for 60–70% of dementia cases^1^. Dementia is characterized by a decline in memory, language, problem solving and other cognitive skills that seriously affects peoples’ everyday activities. The latest report on Alzheimer’s disease facts and figures in 2016 indicates that an estimated 5.4 million Americans suffer from AD, making it the fifth leading cause of death in Americans age ≥65 years. By 2050, one new case of Alzheimer’s is expected to develop every 33 seconds, resulting in nearly 1 million new cases per year^2^.

However, despite substantial intense research, the pathogenic mechanisms underlying AD have not been completely achieved. Several different hallmarks of the disease have been reported including the deposits of amyloid-β (Aβ) around neurons, hyperphosphorylated tau protein, oxidative stress, dyshomeostasis of biometals, chronic nerve inflammation and neuronal cell apoptosis, and low levels of acetylcholine, which reflected the multifactorial nature of this disease^3,4^. Based on these targets, some therapeutic drugs have been developed in clinical or experimental stages, such as cholinesterase inhibitors (increasing acetylcholine level), GSK-3β inhibitors (bringing down hyperphosphorylated tau) and BACE1 inhibitors (reducing Aβ production)^3^. However, current pharmacotherapies only provide temporary symptomatic relief and no completely effective drug is available so far.

Substantial clinical diagnosis and autopsy results demonstrated a disturbance of metal-ion homeostasis in AD, as indicated by high contents of Cu, Zn and Fe present in Aβ plaques, proposing the dysregulation of transition metals played important roles in the pathogenesis of AD^5^. Increasing evidence demonstrated that the zinc (II) and copper (II) interact with Aβ peptides and have an influence on their fibrillization and toxicity^6^. Copper ions entrapped in Aβ fibrils are electrochemically active and can generate ROS, which consequently causes oxidative stress and neurotoxicity^7,8^. Toxicity of the amyloid aggregates was suggested to be depend on copper content^9,10^. On the contrary, Zn^2+^ is redox-inert and plays a protective role in AD by suppressing Cu^2+^-dependent H_2_O_2_ formation from Aβ^11^. Therefore, the homeostasis of transition metals in the brain is closely linked to the development of AD.

Metal-binding proteins exert a vital role in balancing the metal homeostasis in brain, of which metallothioneins-3 (MT3) is the most noticeable. It is a low-molecular-weight (6–7kD), cysteine-rich protein with high metal content (Zn(II), Cu(I)) and predominately expressed within CNS. A body of evidence converged MT3 was downregulated 30% in AD^12–14^, indicating a close association between MT3 and this disease. Originally named neuronal growth inhibitory factor, MT3 was initially shown to inhibit the neurotrophic activity and neuronal growth in the presence of AD brain extracts^13^. So far, some *in vitro* studies demonstrated its multiple functions related to AD. The primary function refers to detoxification and storage of heavy metals and regulation of cellular copper and zinc metabolism. Wing Ming Keung found MT3 antagonized the neurotoxic and neurotrophic effects of Aβ-peptides^15^. In our previous study *in vitro*, we deciphered the roles of single-domain proteins (α/β) and the α–β domain–domain interaction of Zn_7_MT3 to determine the molecular mechanism for protection against the neuronal cytotoxicity of Aβ_1–42_ with copper ions. The two domains of MT3 through the linker Lys-Lys-Ser form a cooperative unit, and each of them is indispensable in conducting its bioactivity. The α–β domain–domain interaction through the linker is critical for its protective role in the brain^16^. Gabriele Meloni revealed the key underlying mechanism on molecular level, that was a metal swap occurring between Aβ-Cu(II) and Zn_7_MT3. This process consequently transformed neurotoxicity Aβ-Cu(II) into ROS-inertia Aβ-Zn(II), brought down the ROS production and rescued cell viability^17^. Besides, Jae-Yong Koh found MT3 exerted a role on modulating the Aβ endocytosis of astrocytes through its effects on actin polymerization, and absence of *Mt3* reduces Aβ uptake in astrocytes^18^. Moreover, Hye Gwang Jeong suggested MT3 regulated the non-amyloidogenic pathway by conversing ADAM10 into its active form, resulting in increased sAPPα and reduced Aβ peptide levels^19^. Although these results above demonstrated the important multiple functions of MT3 to AD, they are *in vitro* studies limited to the molecular or cellular level, failing to reflect how MT3 plays its combined comprehensive role on the typical symptoms of AD. In fact, whether MT3 possesses curative effect to AD can only be further explained by *in vivo* studies, and how it exerts its multiple functions in brain including the influence on metal homeostasis, Aβ deposits and neuron apoptosis should be explored in detail on animal models. However, it is a great pity that systematic *in vivo* studies of MT3 to AD on model animals are very rare so far. Only Juan Hidalgo attempted to explore its role by subcutaneously injecting recombinant Zn_7_MT3 to Tg2576 AD mouse models^20^. The study involved a good amount of valuable research work that broadened our recognition of MT3 on model animals. However, some conclusions are inconsistent with previous *in vitro* studies, which caused the difficulties to draw a definite conclusion. All these reasons above drive us to further elucidate how MT3 exerts its role in AD brain and whether it processes promising effective therapeutic potential.

In this study, the APP/PS1 mice were treated with sustained drug release of Zn_7_MT3 directly to the central nervous system, and the role and molecular mechanism of Zn_7_MT3 to protect against AD mice was investigated systematically. The results demonstrated that Zn_7_MT3 can significantly ameliorate cognitive deficits, improve the morphology and function of hippocampus, regulate metal homeostasis, abolish Aβ plaque load, and reduce oxidative stress and neuronal cell apoptosis in APP/PS1 transgenetic mice. In addition, it has been confirmed that MT3 is penetrable to the blood brain barrier of AD mice. Therefore, Zn_7_MT3 could be an effective AD suppressing agent, and it has potential for applications in Alzheimer’s disease therapy.

## Results

### Chronic Zn7MT3 treatment ameliorates cognitive impairment of APP/PS1 mice

**Figure 1:**
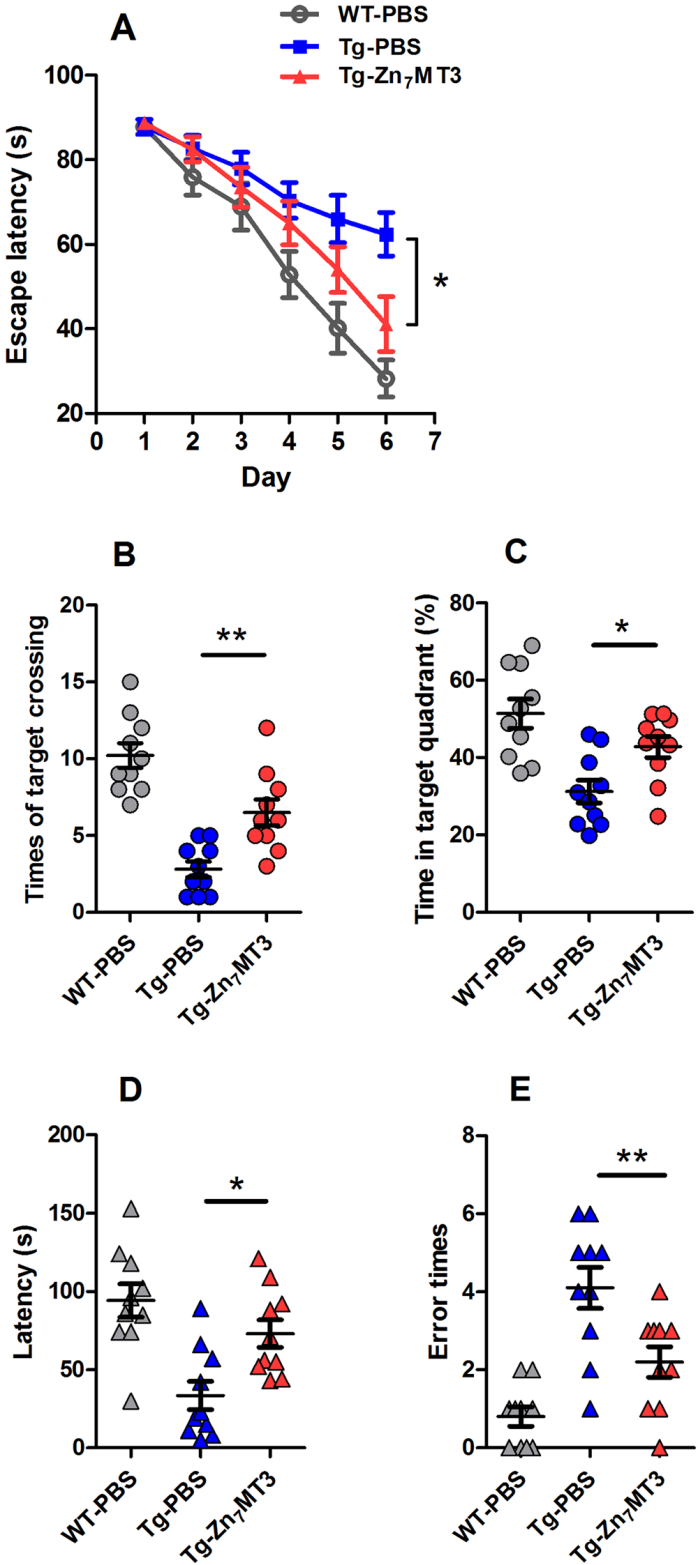
Chronic Zn_7_MT3 administration of APP/PS1 mice leads to attenuation of learning and memory deficits by Morris water maze test and step-down type passive avoidance test. (**A**) In Morris water maze test, all groups showed progressively decreased average escape latencies during 6-day acquisition training. Tg-Zn_7_MT3 mice behaved in significantly reduced average escape latency than Tg-PBS mice in the 6^th^ day. (**B**,**C**) Notable differences could be assessed between Tg-Zn_7_MT3 and Tg-PBS mice in crossing times and time in target quadrant. (**D**,**E**) In step-down type passive avoidance test, Tg-Zn_7_MT3 mice spent significantly more time on blocks and tended to make markedly fewer errors compared to Tg-PBS mice. Data were given as means ± SEM (n = 10 animals per group, **p < 0.01, *p < 0.05). Exact P values were shown in Table S1.

Morris water maze test, the most widely accepted behavioral test of spatial learning and memory was performed in this study to evaluate the potential effect of Zn_7_MT3. During the 6-day acquisition training, all groups demonstrated progressively decreased average escape latencies. Even so, severe cognitive impairment was still observed in APP/PS1 mice, as suggested by notably slower improvement in escape latency in Tg-PBS group. In contrast, Tg-Zn_7_MT3 mice exhibited better performances to locate the hidden platform, as indicated by significantly reduced escape latency on the 6^th^ day (Fig. 1A). Additionally, memory retention was also found to be affected by Zn_7_MT3 as confirmed by probe trial, in which Tg-Zn_7_MT3 mice presented significantly more crossing numbers and time spent in target quadrant (Fig. 1B,C), while the swimming speed of each group were similar (data not shown).

Step-down type passive avoidance test was performed to further confirm the positive effect of Zn_7_MT3 on memory restoration. Consistently, Tg-Zn_7_MT3 mice tended to demonstrate better performances, as indicated by significantly increased latency and reduced error times (Fig. 1D,E). All data in behavioral tests suggested an attenuating effect on cognitive deterioration of Zn_7_MT3 in APP/PS1 mouse model.

### Chronic Zn7MT3 treatment prevents hippocampi from pathmorphological deterioration of APP/PS1 mice

**Figure 2:**
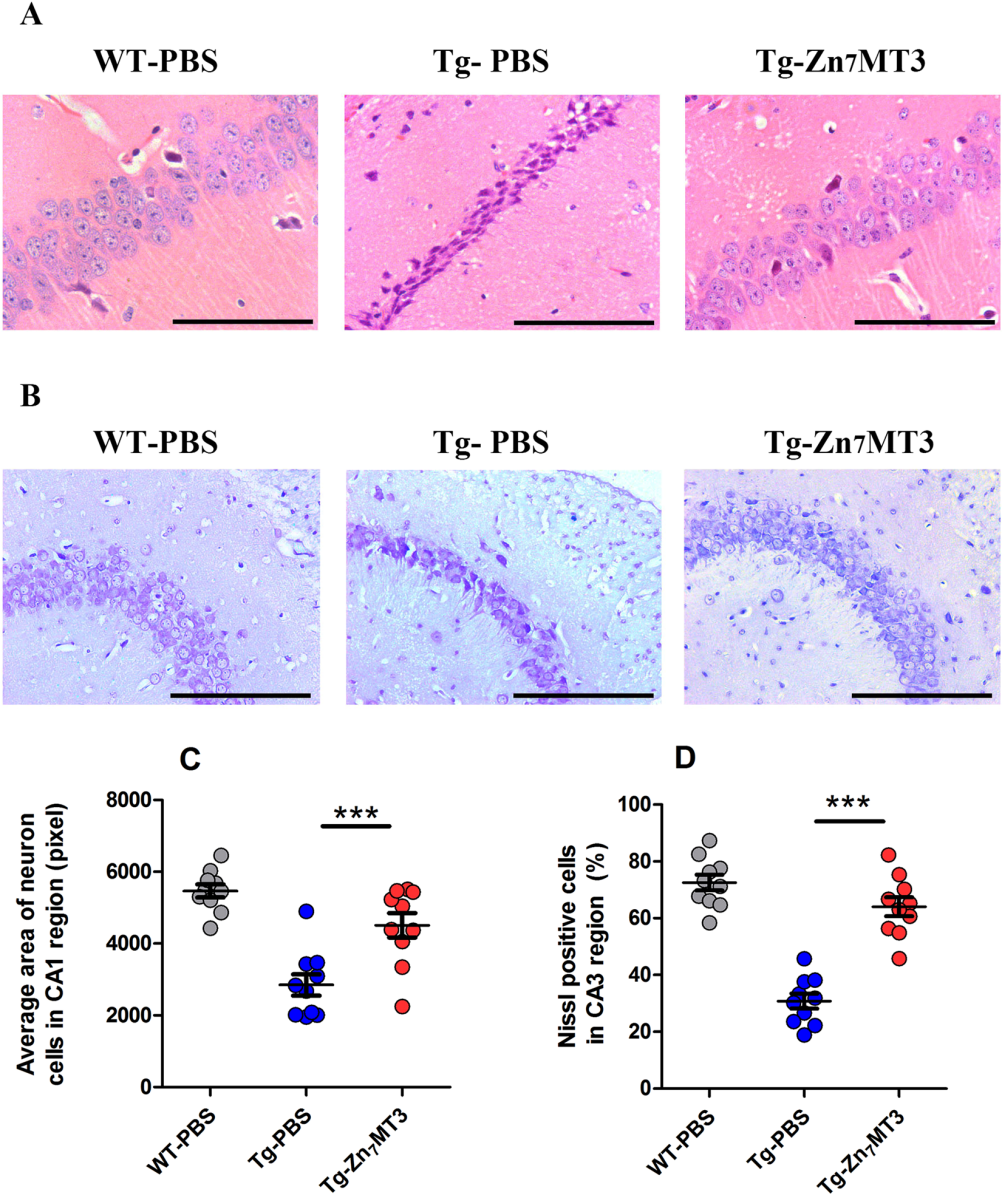
Chronic Zn_7_MT3 treatment results in improved hippocampal histopathological architecture of APP/PS1 mice. (**A,C**) Haematoxylin-Eosin (HE) staining showed the morphology of neurons in CA1 subfield of the hippocampus. Average area of neuron cells in CA1 region notably increased after Zn_7_MT3 treatment. (**B,D**) Nissl staining revealed the features of neurons in CA3 region of the hippocampus. The percentage of Nissl-positive cells markedly recovered after Zn_7_MT3 administration. Data were given as means ± SEM (n = 10 animals per group, ***p < 0.001). Scale bar = 100 μm. Exact P values were shown in Table S2.

Typical AD pathologic features in hippocampus were evaluated. First, the neuron cells in CA1 subfield of the hippocampus were characterized by Haematoxylin-Eosin (HE) staining. Neurons in WT-PBS group exhibited a well-organized architecture with round shape and clear nuclei, while the Tg-PBS mice suffered from poorly-structured neurons with pyramidal appearance and shrinkage nuclei. Zn_7_MT3 was found to abate these pathological features, as demonstrated by recovered neuron cells in better shape and organization in Tg-Zn_7_MT3 mice (Fig. 2A,C). Next, the nissl bodies in CA3 region of the hippocampus were distinguished by Nissl staining. Consistently, severe deformation and disorganization of neuron cells were observed in Tg-PBS mice, with significant reduction of the Nissl-positive cells proportion. After 6-week administration, Tg-Zn_7_MT3 mice exhibited prominent recovery (Fig. 2B,D). These pathmorphological results suggested positive effect of Zn_7_MT3 on hippocampal recovery in APP/PS1 mouse.

### Chronic Zn7MT3 treatment attenuates neuron apoptosis of APP/PS1 mice

**Figure 3:**
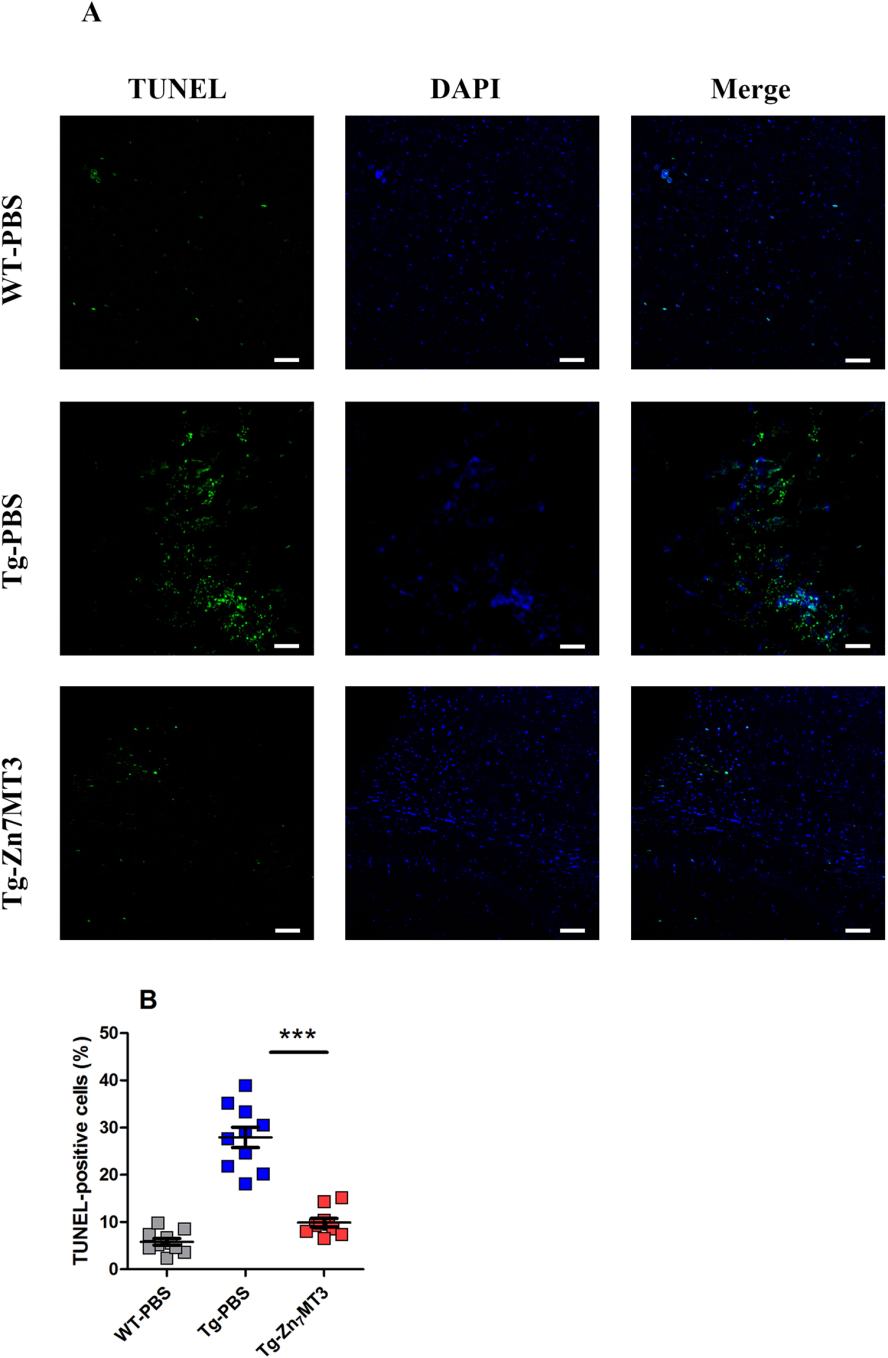
Chronic Zn_7_MT3 treatment attenuates cell apoptosis of APP/PS1 mice. (**A**) TUNEL staining (green) reflected the level of apoptosis while DAPI staining (blue) was used as a counterstain revealing nuclei. (**B**) The proportion of TUNEL-positive cells significantly reduced Zn_7_MT3 treatment. Data were given as means ± SEM (n = 10 animals per group, ***p < 0.001). Scale bar = 100 μm. Exact P values were shown in Table S3.

Cell apoptosis is typical feature of Alzheimer’s disease which contributes to memory dysfunction and tissue damage. To assess whether Zn_7_MT3 produces an effect on cell apoptosis in brain tissues, the terminal deoxynucleotidyl transferase-mediated deoxyuridine triphosphate nick end labeling (TUNEL) staining was implemented. According to the results, Tg-PBS mice exhibited severe apoptosis compared to WT-PBS group, while Tg-Zn_7_MT3 mice suffered from much less apoptosis as revealed by notably reduced TUNEL-positive cells observed (Fig. 3). These results indicated a protective role of Zn_7_MT3 against cell apoptosis in APP/PS1 mice.

### Chronic Zn7MT3 treatment inhibits Aβ plaque deposition of APP/PS1 mice

**Figure 4:**
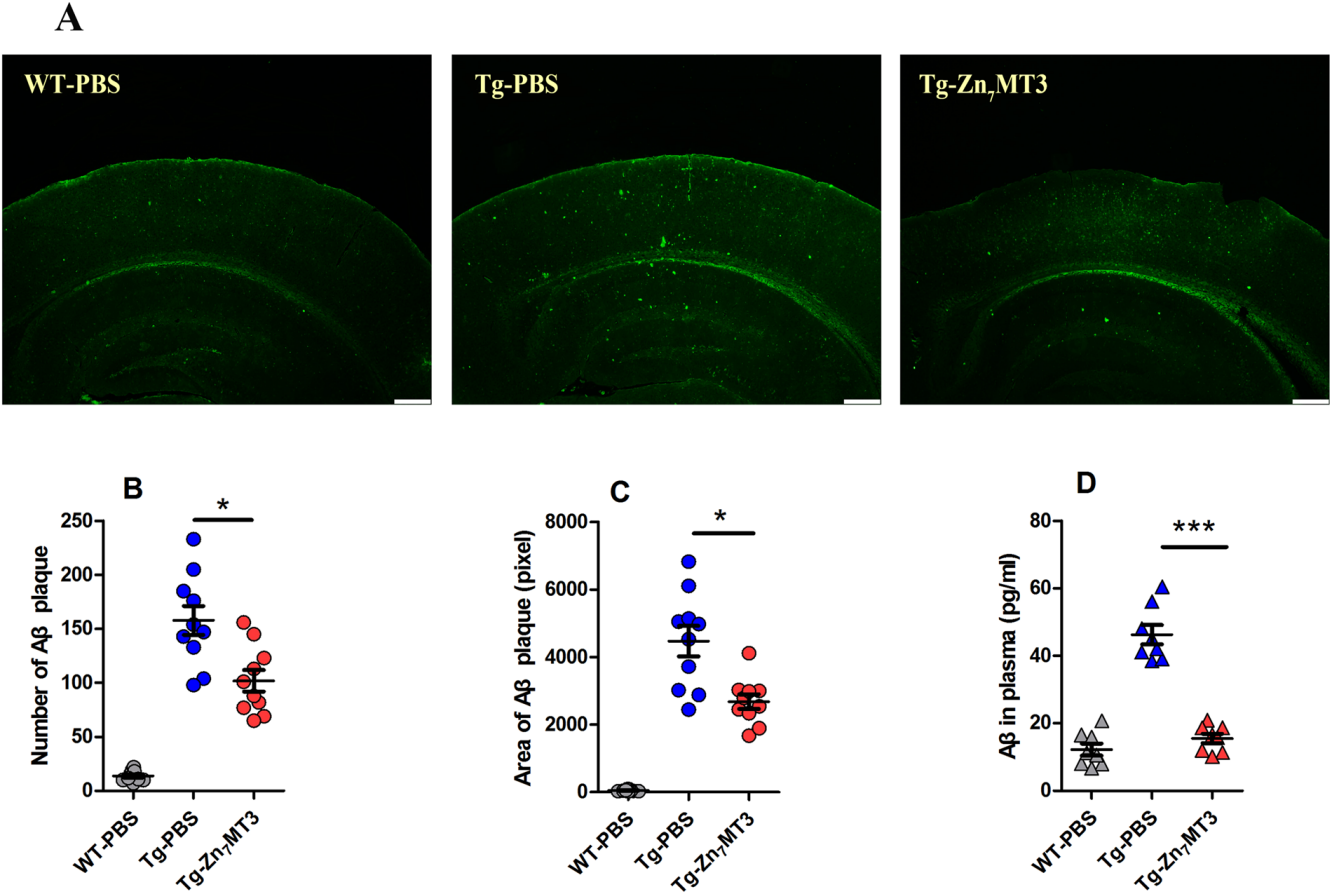
Chronic Zn_7_MT3 treatment abates Aβ plaques in brain tissues and decreases Aβ concentration in plasma of APP/PS1 mice. (**A**) Thioflavin-S staining (green) indicated Aβ plaques aggregated in brain tissues. Scale bar = 500 μm (**B**,**C**) By quantifying number of Aβ plaque and total area of Aβ plaque, significantly differences could been seen between Tg-PBS group and Tg-Zn_7_MT3 mice, which suffered from fewer Aβ deposits. Data were given as means ± SEM (n = 10 animals per group, *p < 0.05). (**D**) Aβ concentration in plasma assessed by ELISA. Tg-Zn_7_MT3 mice had markedly decreased Aβ concentration than Tg-PBS group. Data were given as means ± SEM (n = 8 animals per group, ***p < 0.001). Exact P values were shown in Table S4.

Cerebral Aβ accumulation, as one of the neuropathological hallmarks of AD, is considered the primary causative agent of AD^21^. To evaluate the effect of chronic Zn_7_MT3 administration on Aβ accumulation, 5-month-old APP/PS1 mice, which exhibited moderate levels of preexisting Aβ deposits were chosen to undergo 6-week Zn_7_MT3 treatment. Thioflavin-S staining was performed to distinguish Aβ plaques in brain tissues. The results demonstrated that Zn_7_MT3 notably reduced the Aβ levels, as indicated by much fewer Aβ deposits observed in Tg-Zn_7_MT3 mice (Fig. 4A). By quantifying plaque number and area, significantly differences were observed between Tg-Zn_7_MT3 mice and Tg-PBS group (Fig. 4B,C). Soluble oligomeric aggregates of Aβ are suggested more toxic because soluble Aβ-Cu (II) generates substantial amount of ROS^22^. Therefore, we determined the concentration of soluble Aβ in plasma by ELISA. The results exhibited a sharp decrease in Tg-Zn_7_MT3 mice compared to Tg-PBS group (Fig. 4D). These data suggested a potentially inhibitory effect of Zn_7_MT3 on Aβ deposition.

### Chronic Zn7MT3 treatment alters microdistributions of Cu, Zn and Fe in whole brain of APP/PS1 mice

**Figure 5:**
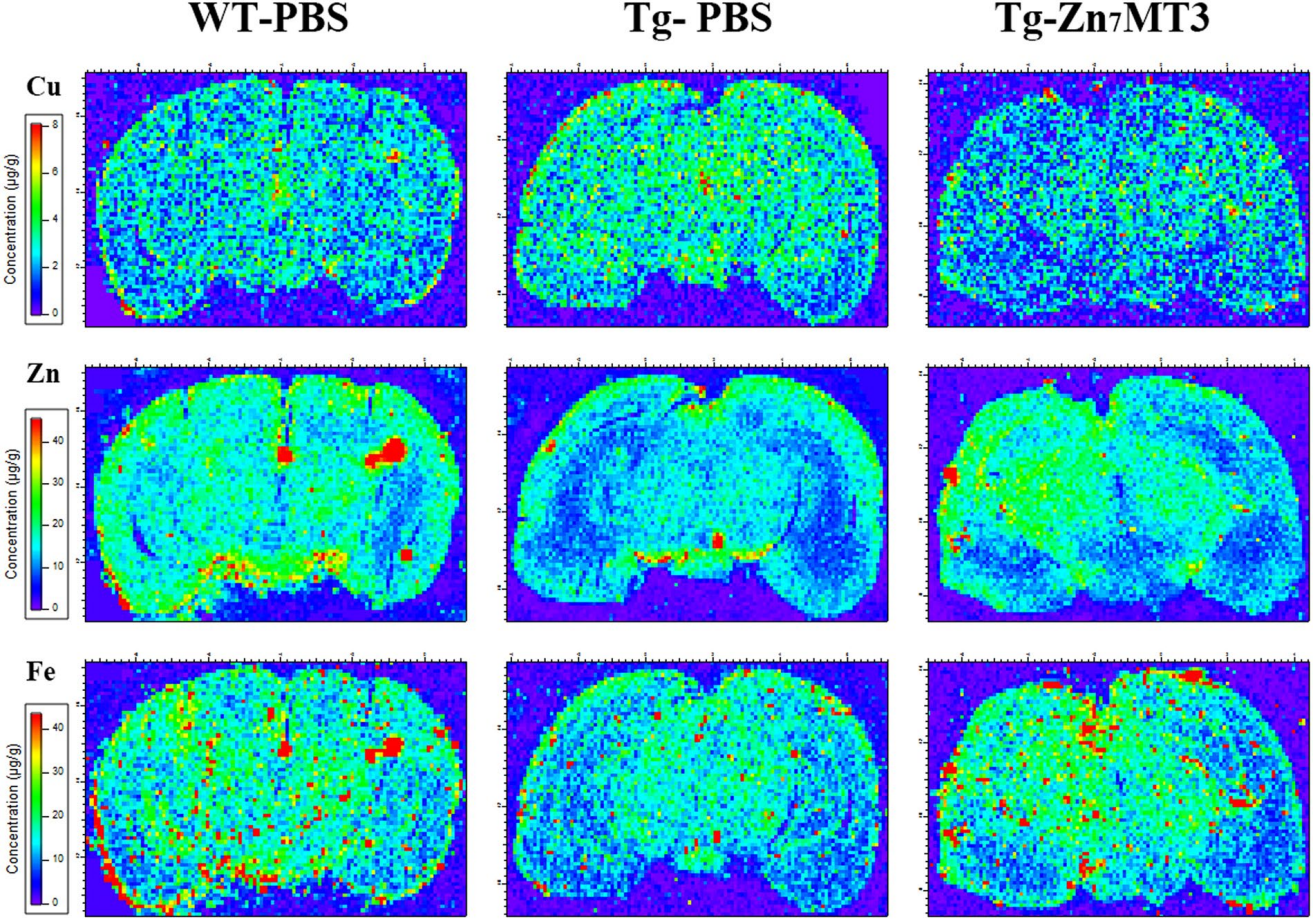
Chronic Zn_7_MT3 treatment influences metal distribution in brain of APP/PS1 mice. Images of Cu, Zn and Fe via synchrotron radiation micro beam X-ray fluorescence (SR-μXRF) manifested a regulating role of Zn_7_MT3 on metal distribution.

**Figure 6:**
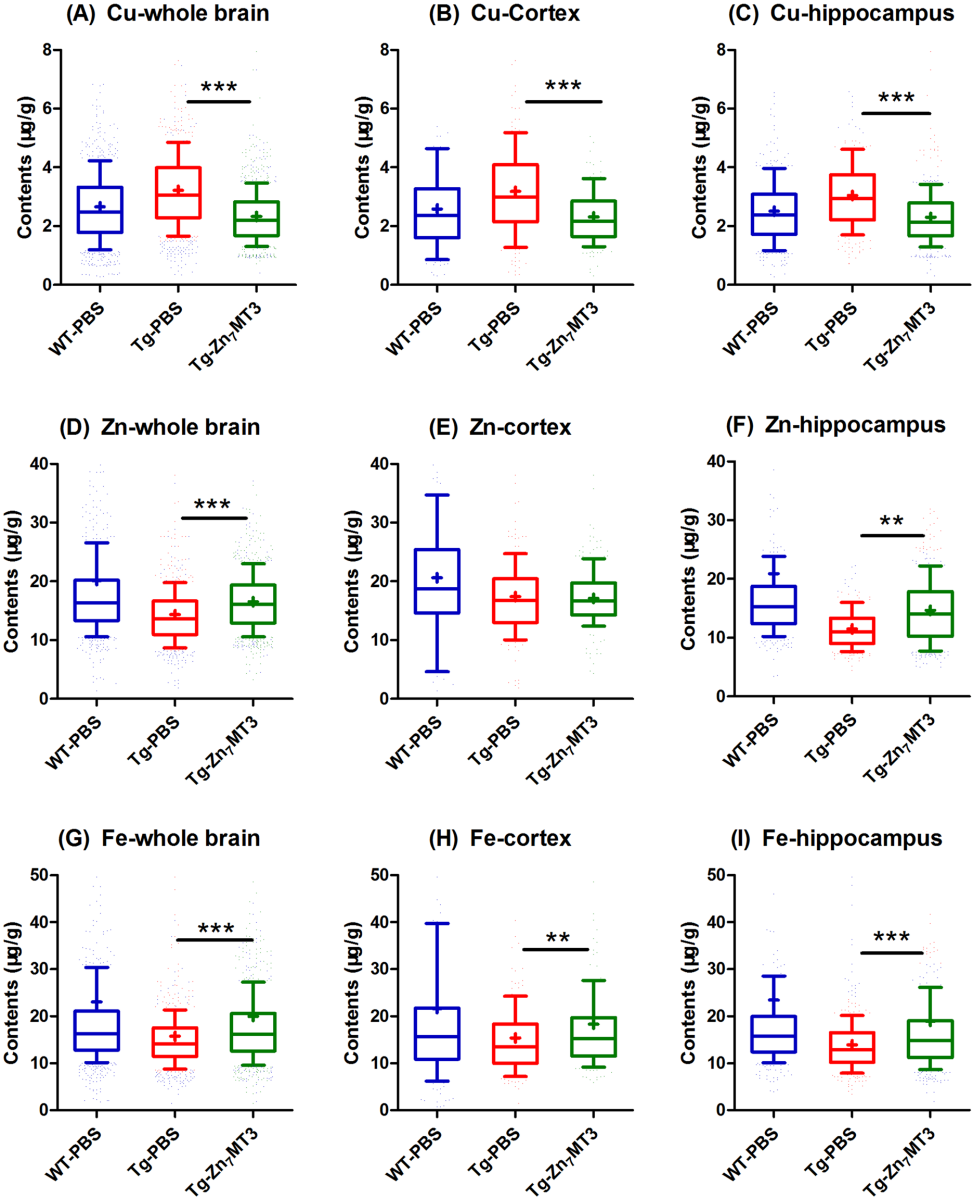
Quantitative analysis reveals alterations of Cu, Zn and Fe among different groups in whole brains, cortexes or hippocampi. Data were given as means ± SEM (n = 1600–2000, 350–400 and 600–800 scanned spots for whole brain, cortex and hippocampus, respectively, ***p < 0.001, **p < 0.01, *p < 0.05). Each group included 6 animals. Exact P values were shown in Table S5.

Alzheimer’s disease is associated with dysregulated metals homeostatsis^23–25^. MT3, as a metal-binding protein, is considered to have function of storage and regulation of heavy metals. In our study, synchrotron radiation micro beam X-ray fluorescence (SR-μXRF) was involved to directly and visually assess the metal regulating effect of Zn_7_MT3 in brain tissues of APP/PS1 mice. The images demonstrated that biometal distributional abnormalities existed in Tg-PBS mice as opposed to the WT-PBS mice, as indicted by elevated Cu level and reduced Zn and Fe levels in whole brain as well as varied contents in cortex and hippocampus (Fig. 5; Fig. S1). This tendency of disorder metal distributions was partially reversed by chronic Zn_7_MT3 treatment. In the whole brain of Tg-MT3 mice, Cu level exhibited a notable decrease while the contents of Zn and Fe showed significant elevation (Fig. 6A,D,G). In WT-PBS mice, Cu in hippocampus and cortex are at similar level, while in Tg-PBS mice, Cu content in cortex is significantly higher than in hippocampus. Zn_7_MT3 treatment resulted in reduced Cu content to the resembling level in these two regions (Fig. 6C; Fig. S1C). Zn in Tg-PBS mice distributed at significantly higher level in cortex than in hippocampus while no notable difference was observed in WT-PBS mice. Zn_7_MT3 treatment compensated Zn loss primarily in hippocampus despite of the unchanged inhomogeneous distribution in Tg-MT3 mice (Fig. 6F; Fig. S1F). Fe concentrated in cortex more intensively than in hippocampus in Tg-PBS mice, while the contents were of no obvious difference in WT-PBS mice. Zn_7_MT3 treatment partially abated such abnormality by notably increasing Fe level in hippocampus and narrowing the gap between hippocampus and cortex in Tg-MT3 mice (Fig. 6I; Fig. S1I). These results exhibited a positive metal regulating effect of Zn_7_MT3 to AD mice.

### Chronic Zn7MT3 treatment brings in reduction of oxidative stress

**Figure 7:**
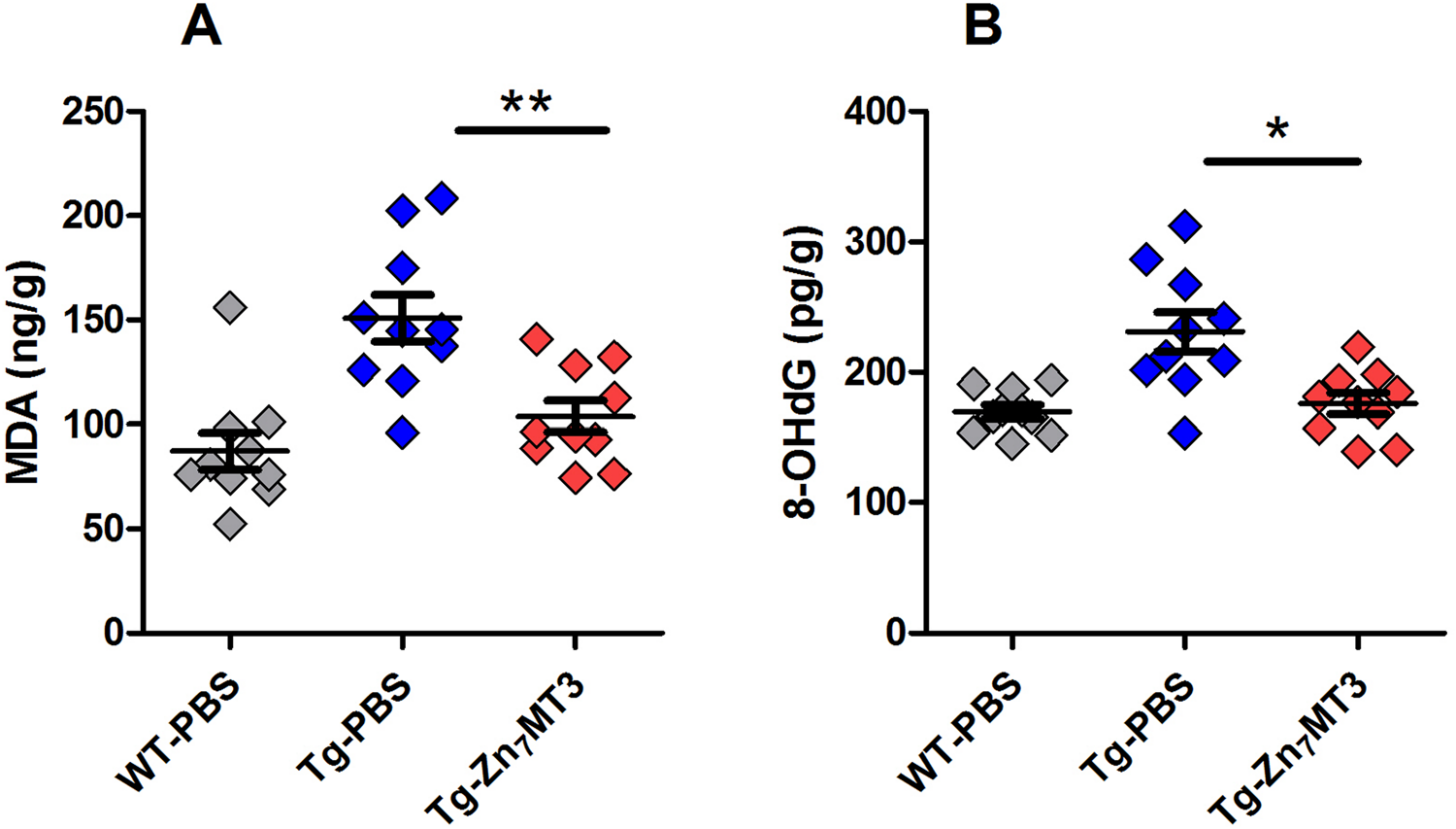
Chronic Zn_7_MT3 treatment reduces oxidative stress in brain of APP/PS1 mice. (**A**) Concentration of MDA in brain tissue assessed by ELISA. (**B**) Concentration of 8-OHdG in brain tissue determined by ELISA. Data were given as means ± SEM (n = 10 animals per group, **p < 0.01, *p < 0.05). Exact P values were shown in Table S6.

Oxidative stress (OxS) is an early event in AD pathogenesis, which can precede the formation of Aβ deposits. Quantification of the byproducts of oxidative biomolecular damage is widely adopted to evaluate OxS level, since the direct measurement of reactive species is challenging and infeasible for practical applications. In our study, malondialdehyde (MDA) and hydroxy-2′-deoxyguanosine (8-OHdG), which are typical markers of lipid peroxidation and nucleic acid oxidation respectively, were assessed by ELISA. The results demonstrated that both MDA and 8-OHdG levels significantly reduced in Tg-Zn_7_MT3 mice compared to Tg-PBS group, revealing a special effect of Zn_7_MT3 to abolish ROS and abate oxidative stress (Fig. 7).

### MT3 is penetrable to the blood brain barrier of AD

**Figure 8:**
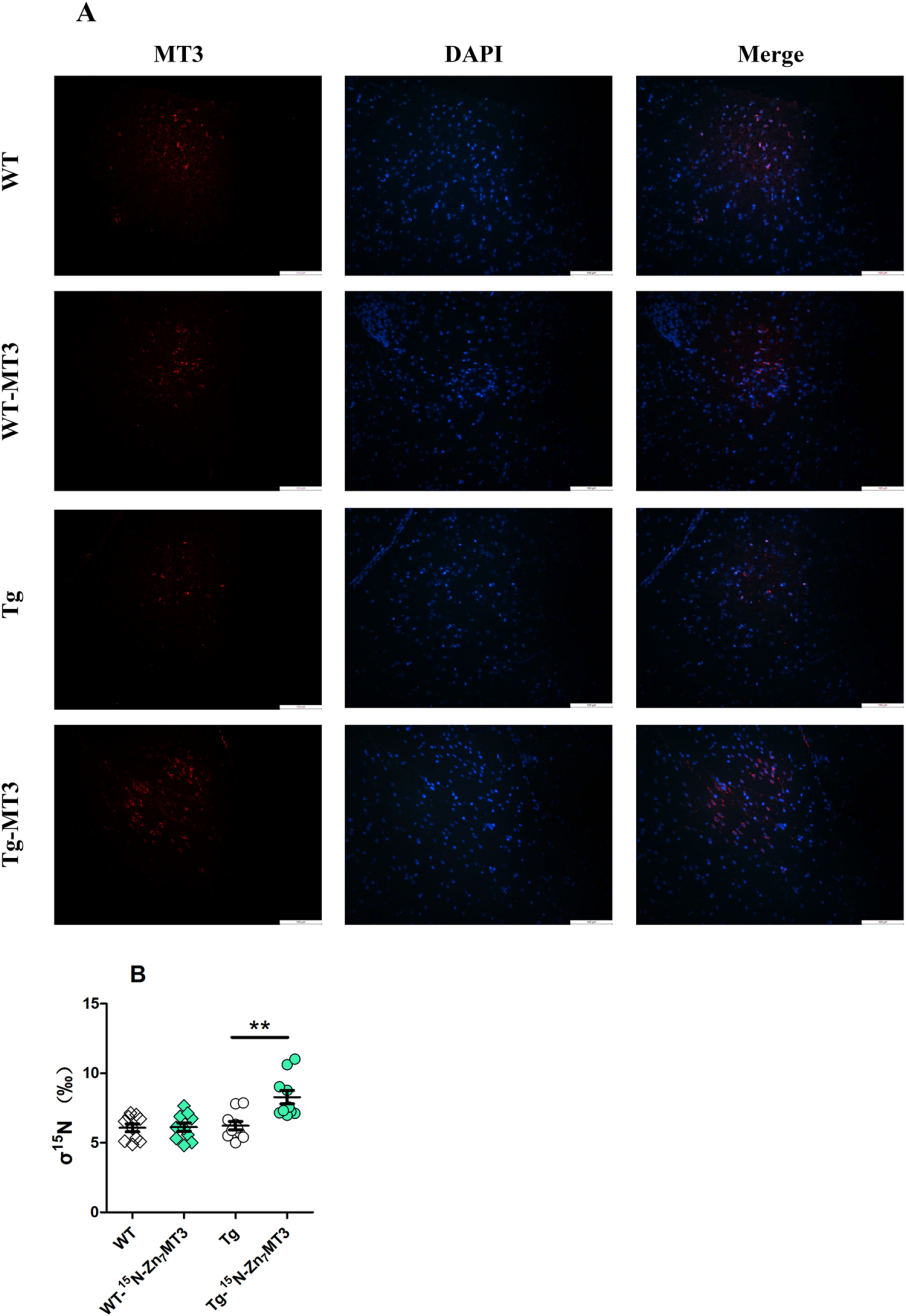
MT3 is penetrable to the BBB of AD model mice. After intravenously injected with ^15^N-Zn_7_MT3, increased Zn_7_MT3 immunofluorescence staining (**A**) as well as notably higher ^15^N stable isotope abundance (**B**) were observed. Data were given as means ± SEM (n = 10 animals per group, **p < 0.01). Scale bar = 100 μm. Exact P values were shown in Table S7.

Metallothionein protein was proven to be incapable to cross the intact blood brain barrier (BBB)^26^. However, when the BBB was damaged, intraperitoneally-administered MT protein could enter the central nervous system^27,28^. To investigate the permeability of MT3 protein to the BBB of AD, we intravenously injected ^15^N labeled MT3 to AD model mice and examined the variation of ^15^N isotopic abundance in brain tissue. Meanwhile, immunofluorescence was performed to further confirm the results. We found that both the ^15^N abundance and immunofluorescence staining suggested notable increases in the group injected with MT3, which revealed a penetrable characteristic of MT3 to BBB of AD model mice (Fig. 8).

## Discussion

In our experiment, intraventricular delivery was chosen. In this way, we excluded the potential impediment of the BBB, ensured the arrival of Zn_7_MT3 to CNS and guaranteed the physiological changes we observed originated from the effect in brain rather than the peripheral blood circulation. Although we found MT3 could partially cross the disrupted BBB of AD mouse at the end of our study, little was know about its stability *in vivo* and biological availability. Moreover, considering the previous *in vivo* study by injecting Zn_7_MT3 subcutaneously^20^ exhibited some inconsistent results with most *in vitro* studies, we selected intraventricular delivery strategy. On the other hand, we innovatively fused Smt3 tag instead of GST tag used in previous reports^29^. Consequently, MT3 of exactly 68 amino acids could be produced by SUMO protease digestion. These methods adopted were helpful for diminishing interference factors.

Substantial evidence proved that Alzheimer’s disease is closely associated with dysregulated metals homeostasis and abnormal metal-protein interactions^23–25^. MT3 was proposed to have physiological functions including detoxification and storage of heavy metals and regulation of cellular copper and zinc metabolism. In our study, we for the first time involved Synchrotron radiation micro beam X-ray fluorescence (SR-μXRF) to directly monitor and visually assess how Zn_7_MT3 exerted its metal-regulating role in brain of APP/PS1 mouse model. The results showed that chronic Zn_7_MT3 treatment rescued the deteriorating imbalance of microdistributions of Zn, Cu and Fe in whole brain of APP/PS1 mice. Zinc is necessary for brain development and physiology. Neurological complaints associated with Zinc deficiency such as learning, memory, and emotional stability are related to functions of brain structures normally rich in this metal, such as hippocampus, amygdale, and neocortex, among which the neocortical region most prone to AD^30^. Ventriglia *et al*. performed meta-analyses of 27 studies on Zinc levels in AD from 1983 to 2014, and the results supported the hypothesis that lower Zinc level was associated with AD^31^. A growing body of evidence suggests that a deficiency, rather than an excess, of zinc leads to an increased risk for the development of neurological disorders and memory deficits^32^. Some proteins, which are involved in the uptake, excretion, and intracellular storage/trafficking of zinc such as MTs, have been reported decreased in both aging and AD^32^. This can be an explanation for zinc deficiency. A consistent trend of decreased Zn level was found in our APP/PS1 model mice, especially in the hippocampus region. Our results revealed that Zn_7_MT3, as a Zinc pool, partially rescued the deteriorating Zn-loss trend as indicated by notably compensating Zinc content in hippocampus. Various studies, including animal testing and clinical trials, have investigated the effect of zinc supplementation on cognitive function in elderly subjects^33–38^. Among these, Corona *et al*. studied the effect of dietary zinc supplementation in a transgenic mouse model of AD and the results showed that controlling brain zinc homeostasis was beneficial in delaying hippocampal-dependent memory deficits and strongly reducing both Aβ and tau pathology^39^. Concerning human, the Zincage study demonstrated that the subjects’ cognitive functions were decreased related to decreased zinc levels^40,41^, and the Zenith study reported a certain beneficial effect of 15 and 30 mg/d of zinc supplementation^42^. Zinc is a copper competitor in intestinal absorption, and some investigations evidenced to advocated zinc therapy as a safe an effective treatment of removing copper and free copper intoxication^43,44^. Zn_7_MT3, which plays a role as zinc pool, can be a potential alternative of zinc supplement, since it can re-establish the balanced zinc homeostasis according to our study.

Besides, the exciting role of Zn_7_MT3 is much more than simple zinc supplement, but what is more important, lies in abating the dysregulated metals homeostasis in AD, including Cu and Fe according to our study. During the last two decades, high free copper has shown promising evidence in favor of association with the etiology of AD^45^. It catalyzes formation of amyloid in plaques and oxidative stress causing neurodegeneration^46^. In cell cultures, Aβ toxicity has been shown to depend upon its binding to Cu^47,48^, via the generation of radicals^22,49^. Moreover, a faster rate of cognitive decline was reported among persons with higher copper intake^50^. Bucossi *et al*. applied meta-analysis to a selection of 26 studies published in the literature and confirmed elevated Cu levels in AD^51^. Our APP/PS1 model mice exhibited a consistent elevation in Cu level in the whole brain. MT3, as main metallothioneins isoform in central nerve system, binds zinc and copper and plays an important role in the homeostasis of zinc (II) and copper (I) absorption and storage^52^. Some studies found metallothioneins are involved in the process of copper detoxification^53^. *In Vitro*, Zn_7_MT3 was confirmed to remove copper from soluble and aggregated Aβ–Cu (II) through a metal swap, thus can transform copper-induced Aβ aggregation, which relates to ROS production and neurotoxicity, into zinc-induced Aβ aggregation, which is considered to be neuroprotective^11,17^. Such mechanism provided an explanation to our *in vivo* results, in which chronic Zn_7_MT3 treatment resulted in reduced Cu level in whole brain of model mice. On the other hand, iron is the most abundant transition metal in the brain. Many studies suggested that iron protected the oxidative damage by combing with different enzymes due to its redox activity^54^. Iron is also well known to acts as a cofactor in the synthesis of neurotransmitter in brain^55^. Evidence indicated that the cortical area was the main plaque deposition region and iron was enriched in plaques. However, in our study, APP/PS1 model mice suggested a decreased iron level in the cortex. The low Fe content might reveal defective metal transport in some micro-regions of the AD brain as supported by some previous reports^56,57^. Two proteins, ferritin and transferrin, are most involved in iron regulation for storage and mobilization respectively. In Alzheimer’s disease, transferrin is decreased in the various cerebral cortical regions particularly in the white matter, indicating a reduced mobility and subsequent utilization of iron in the brain^56^. Our study observed that iron content increased in brain and its homeostasis recovered from imbalance after chronic Zn_7_MT3 treatment.

Since Zn_7_MT3 rescued metal dyshomeostasis in AD brain, we further probe the downstream influences on oxidative stress. A large body of evidence from *in vitro* and animal studies suggested that oxidative stress (OxS) was an early feature in AD pathogenesis^11,23,58–60^. Metal dyshomeostasis are closely related with the elevated reactive oxygen species (ROS) level, which is one of the primary oxidants that leads to damage^4,61^. Reduced transition metals (Cu^+^ or Fe^2+^) act with Aβ oligomers and consequently generate oxygen peroxide and hydroxyl radicals. We evaluated the two common markers of OxS, MDA and 8-OHdG. Consistent with a number of previous studies^4^, model mice showed increased levels of MDA and 8-OHG in brain, while chronic Zn_7_MT3 treatment resulted in decreased OxS level, which we considered was an accompanying outcome of re-established metal homeostasis. Since Aβ-Cu(II) generated ROS through the redox cycling of copper, excess Cu in brain is neurotoxicity^22^. In contrast, Aβ-Zn(II) is considered to be neuroprotective due to its ROS inertia^11,62^. Zn_7_MT3 treatment could quench ROS by metal swap with Aβ-Cu^17^, help to recover Cu and Zn homeostatsis and consequently abate oxidative stress.

The accumulation of Aβ in brain is one of the hallmarks of AD. Our study discovered that both Aβ deposits in brain and soluble Aβ in plasma exhibited significant reductions after Zn_7_MT3 administration. Since metal swap did not impede Aβ aggregation but only altered the nature of the final product^17^, we considered that decreased Aβ was associated with astrocytes’ endocytosis and reduced OxS. The extracelluar Aβ level is determined by its secretion rate and the rate of its clearance through endocytosis^63,64^. Astrocytes were reported to be the main contributors to the clearance and degradation of Aβ peptide^65,66^. MT3 was suggested to modulate the Aβ endocytosis of astrocytes through its effects on actin polymerization^18^. Therefore, supplement of Zn_7_MT3 is likely to advance Aβ uptake and degradation in astrocytes. On the other hand, OxS was indicated to play a role in the generation and deposition of Aβ in brain^67^. Accumulating experimental evidence from transgenic animal models suggests that OxS significantly increases the catalytic activity of β- and γ-secretases, which in turn augments Aβ production^60,68^. Consequently, decreased OxS level could cut down the Aβ generation. Moreover, we examined the APP expression level by RT-PCR, and results showed a downregulated trend after Zn_7_MT3 treatment, which we inferred was another contributor to reduced Aβ in brain (Fig. S2).

These combined effects above of re-established metal homeostasis, reduced OxS level and decreased toxic Aβ peptide, consequently led to attenuated neuron apoptosis and abated typical pathological characteristics in hippocampal region. Therefore, it could be concluded that Zn_7_MT3 not only functioned as Zinc supplement as a Zn pool, but what is of great significance is its unique multiple roles for alleviate the abnormalities in AD. These functions finally resulted in a positive presentation in behavioral experiments, suggesting Zn_7_MT3 played a role on reversing cognitive deficits. Comparing with previous study^20^, our results found some inconsistencies, mainly in Aβ quantity in brain and Morris water maze test. These discrepancies might be attributed to different animal models, way of administration or drug dose.

Previous study has proven that metallothionein protein was incapable to cross the intact blood brain barrier (BBB)^26^. However, when the BBB was damaged by induction of EAE^27^, or traumatic brain lesion^28^, intraperitoneally-administered MT protein could enter the central nervous system (CNS). In our study, by labeling MT3 with ^15^N stable isotope as well as by performing immunofluorescence, we for the first time proved MT3 was able to cross the BBB of AD model mice, which was possibly attributable to the damaged or compromising BBB in AD animals. Based on such permeability and all the positive functions, Zn_7_MT3 was potentiated to be an effective therapeutic agent to AD.

## Materials and Methods

### Ethics statement

This study has been approved by the Institutional Animal Care and Use Committee of Fudan University. All animal experiments were carried out in accordance with the animal experimental guidelines set by National Institutes of Health Guide for the Care and Use of Laboratory Animals, and all efforts were made to minimize the animals’ suffering.

### Preparation of recombinant human Zn7MT3

The cDNA sequence of human metallothionein-3 was integrated into plasmid MBPHT-mCherry 2 vector by restriction enzymes *BamHI* and *HindIII*. Fusion protein MBP-smt3-mt3, efficiently expressed via *Escherichia coli* strain BL21(DE3) pLys on LB medium and purified by Ni-NTA column, was digestion by SUMO protease to produce MT3. Pure MT3 was obtained after removing MBP-smt3 tag and His-SUMO protease by Ni-NTA column and Superdex G75 size exclusion chromatography successively. The purity and correctness of the recombinant proteins were confirmed by SDS-PAGE and ESI-MS analysis. The fully Zn(II)-loaded form Zn_7_MT3 was prepared by metal reconstitution^69^. First, Apo-MT3 was obtained by removing metals from acidic MT3 protein solution via Superdex G25 gel filtration. Next, Apo-MT3 was immediately moved into anaerobic glove box and incubated with ZnCl_2_ solution (seven-fold molar concentration of protein), then the pH was slowly adjusted to 8.0~9.0 by dripping 2 M Tris. During the whole metal reconstitution process, 50 mM DTT in protein solution was contained and all solutions in anaerobic glove box were deaerated in advance. Protein concentration was measured via photometric sulfhydryl groups (CysSH) quantification upon their reaction with 2,2-dithiopyridine in 0.2 M sodium acetate/1 mM EDTA (pH 4.0) using ε_343_ of 7.06 × 10^3^ M^−1^ ^70^. Zinc content was determined by Hitachi P-4010 inductively coupled plasma optical emission spectrometer (ICP-OES) at Instrumental Analysis Center of Fudan University, Shanghai. Zinc-to-protein ration was calculated to evaluate zinc reconstitution completion of MT3, and in all cases, the ratio of 7.0 ± 0.2 was obtained. Endotoxin removal from protein solution was achieved primarily by 10kD ultrafiltration membrane (Milipore), further followed by polymyxin B affinity column in ToxinEraser^TM^ Endotoxin Removal Kit (GenScript). Endotoxin content was semi-quantified via ToxinSensor^TM^ Gel Clot Endotoxin Assay Kit (GenScript). Finally, protein solution of reduced endotoxin was lyophilized and stored in −20 °C for use. ^15^N-Zn_7_MT3 was prepared following the same procedure above, except for the minimal culture medium containing ^15^NH_4_Cl used.

### Transgenic mice and treatment

APPswe/PSEN1dE9 (APP/PS1) double transgenic mice (approx. 5 months old, weighing 20–25 g) on a C57BL/6 background used in this study were obtained from Shanghai Research Center for Model Organisms (Shanghai, China). Mice were housed in a 12-h light/12-h dark cycle with free access to autoclaved water, diet at constant room temperature of 25 °C.

In order to ensure the drug’s arrival and local effects in brain tissues, Zn_7_MT3 was directly and continuously delivered to animals’ central nervous system (CNS) using mini-Osmotic pumps (Model 2006; Alzet) and brain infusion kit 3(Alzet). Each pump, filled with 2 mg Zn_7_MT3 (dissolved in 200 μl PBS) or vehicle (PBS), was connected to brain infusion cannula with catheter tube and carefully implanted subcutaneously in the mouse following the manufacturer’s surgical procedures. The stereotaxic instrument was involved to place cannula at accurate position in animals’ cerebral ventricles (lateral 1.0 mm, posterior 0.4 mm to bregma as the zero point). Animals were randomly assigned into three groups: APP/PS1 mice treated with Zn_7_MT3, APP/PS1 mice treated with vehicle (PBS), and wild-type mice treated with vehicle (PBS). Continuous drug delivery at the dose of 50 μg/day/mouse lasted for six weeks.

### Behavioral characterization

Morris water maze test and step-down type passive avoidance test were performed to evaluate learning and memory ability of treated APP/PS1 mice after six-week administration. The Morris water maze involved in this test was a circular tank of 120 cm diameter and 50 cm height filled to a depth of 25 cm opaque water (23–25 °C). The surface area of the tank was divided into four equal quadrants and an escape platform (10 cm diameter) was fixed submerged 2 cm below water surface. The mouse was trained to find the hidden platform via only distal spatial cues available in the testing room. During the acquisition training period, each mouse was released into the tank from designed starting point and given 90 s to find the platform. Then the mice were either allowed to stay on platform for 10 s (if they reached it themselves) or manually guided to remain on platform for the same time (if they failed). The animals were trained for four trails (each from different quadrant) with a 15-min inter-trial interval per day for 6 consecutive days. Their movements were imaged by camera and escape latency was recorded. A probe trial was performed 1d after 6d training trial to assess the memory consolidation. In this trial, the platform was removed from the tank, and the mice were released from the quadrant opposite to previous platform location to swim freely for 180 s. Time spent in the target quadrant and the numbers of crossing previous platform location were recorded to evaluate spatial memory.

In step-down type passive avoidance test, mice were placed into the apparatus of a box with a wooden block placed in the corner. When electric currents (0.5 mA for 2 s) were delivered, the mice would jump onto the block to avoid the electrical foot shock. Once the mice stepped down from the block, they would be subjected to an electrical shock again through the grid floor. Mice were received training trial for 5 min after they placed into the box for five-minute adaption. After 24-hour interval test trial was performed, in which animals were placed on the block and latency to step down on the grid with all four paws for the first time as well as the number of errors subjected to shocks within 5 min were measured as learning performances.

### Histochemistry and histopathology

Animals were anesthetized with intraperitioneal injection of chloralhydrate, perfused with 0.9% saline, and sacrificed by decapitation. Brain tissues were fixed in 4% paraformaldehyde for 24 h, paraffin-embedded and cut coronally into 5 μm-thick sections. Before staining, sections were deparaffined in xylene, rehydrated in a graded series of ethanol, and rinsed in PBS.

Amyloid plaques were characterized by staining with Thioflavin-S. Brain sections were treated with 0.1% Thioflavin-S in 50% ethanol for 10 min at room temperature, washed with 50% ethanol for three times, and sealed with a cover glass using mounting solution. All stained sections were examined by laser scanning confocal microscopy (Leica, Germany). The plaque number and plaque area were evaluated using Image Pro Plus Software.

Hematoxylin and eosin (HE) staining was done to evaluate neuronal cell features, and cresyl violet staining was performed to characterize Nissl granules. Photographs were taken under a light microscope (Leica, Germany). Average areas of neuron cells and number of Nissl positive/negative cells were assessed by Image Pro Plus Software.

### Immunofluorescence

Brain sections were deparaffined in xylene, rehydrated in a graded series of ethanol, and rinsed in PBS. After being blocked in 5% BSA, the slides were incubated with primary antibodies MT3 (GeneTex, GTX60188) with a dilution of 1:100 at 4 °C overnight, followed by staining with secondary antibody at 1:100 for 1 h at room temperature. The sections were examined on laser scanning confocal microscopy (Leica, Germany).

### TUNEL assay of neurons apoptosis

Apoptotic neurons were determined by terminal deoxynucleotidyl transferase (TdT)-mediated dUTP-biotin nick-end labeling (TUNEL) staining DNA fragmentation assay kit. After being deparaffined, rehydrated and rinsed in PBS, brain tissue sections were incubated with proteinase K for 20 minutes, and then with TUNEL reaction mixture containing label and enzyme solution away from light. Slides were examined on laser scanning confocal microscopy (Leica, Germany). The percentage of the TUNEL-positive cells was evaluated.

### Enzyme-linked immunosorbent assay (ELISA)

Determination of Aβ_1–42_, Malondialdehyde (MDA) and 8-Hydroxydeoxyguanosine (8-OHdG) in plasma or brain tissues were performed using a competitive ELISA commercial kit (Cloud-clone) according to the manufacturer’s instructions. Fresh mice blood serum samples were collected into tubes containing heparin at ophthalmic vein and centrifuged for 20 minutes at 1000 g after being kept at room temperature for 2 h. Protein extraction were done in RIPA buffer (Sigma-Aldrich) after frozen brain samples were weighed with a precision scale and homogenized. DNA extraction was performed using a rapid animal genomic DNA isolation kit (Sangon Biotech).

### Quantitative Real-Time PCR (RT-PCR)

Total RNA was extracted from frozen brain tissues with TRIzol Reagent (Takara, Japan) and reversed-transcribed to cDNA with Prime Script TM RT Master Mix (Takara, Japan) according to manufacturer’s instructions. Real-time PCR was performed on Applied 21 Biosystems 7500 (Life Technologies Corporations,Carlsbad, CA, USA) according to the procedure of denaturation at 95 °C for 30 s followed by 40 cycles of denaturation (95 °C, 5 s), annealing (55 °C, 30 s), and extension (72 °C, 30 s). Relative expression changes were analyzed using ΔΔCt method where the amount of target was normalized to the amount of endogenous control (GAPDH). The specific primer sequences are as follows:

GAPDH: Forward: 5′-CCAGCCCAGCAAGGATACTG-3′

Reverse: 5′-GGTATTCGAGAGAAGGGAGGGC-3′

APP: Forward: 5′-CCGTTGCCTAGTTGGTGAGT-3′

Reverse: 5′-GCTCTTCTCGCTGCATGTC-3′

### Synchrotron radiation micro beam X-ray fluorescence (SR-μXRF) analysis

The distributions of Cu, Zn and Fe in the mouse brain section were analyzed via SR-μXRF at beamline BL15U of Shanghai Synchrotron Radiation Facility (SSRF, Shanghai, China) as previously described^57^. Briefly, mouse brain specimens were frozen and cut sagittally into 40 μm sections and dried on 3 μm–thick Mylar films (polycarbonate film) at room temperature. The continuous synchrotron X-rays were monochromatized by a Si(111) double crystal. A monochromatic X-ray beam with photon energy of 10 keV was used to excite the samples. The cross-section of the beam irradiating on the samples was adjusted to about 60 × 60 μm^2^ and scanned in a 60-μm step for both the x- and y-directions, with 1 second irradiation of each spot. Fluorescence counts of the metals for each pixel were extracted from energy dispersive spectra using the software GeoPIXE (CSIRO, Australia). The intensity maps of target metals were imaged using the software Igor Pro (WaveMetrics, USA). Bovine liver (NIST 1577a) was chosen as the standard reference materials. Compton scattering in XRF spectra was used as an internal standard to compensate the differences in thickness and density of thin bio-tissue sections. The relative ratios of elemental sensitivities to Compton scattering obtained from matrix-matched standard reference materials were used for the calculation of metal concentrations in the bio-tissue sections^57^. Data representing each scanned spot for specific brain region were randomly selected for statistical analysis with exclusion of exotic data. No less than 350 and 600 spots were chosen for cortex and hippocampus region, respectively.

### ^15^N stable isotope ratio analysis

To test the blood brain barrier (BBB) permeability of MT3, each transgenic mouse was intravenously injected with 1.5 mg ^15^N-Zn_7_MT3 and sacrificed after 1 hour. Brain tissues were homogenized and lyophilized, and the ^15^N isotope ratio in brain samples were determined by Elemental analysis-stable isotope ratio mass spectrometer (IRMS) at Instrumental Analysis Center of Shanghai Jiaotong University.

### Statistical analysis

All experimental data were presented as mean ± standard error of the mean (SEM). Significance levels were given as follows: ***P < 0.001; **P < 0.01; *P < 0.05. Statistical analysis was performed using one-way ANOVA followed by Tukey’s or Dunnett’s test for post hoc analysis to identify significant effects. All statistics were performed using SPSS 20.0 software and graphs were generated by GraphPad Prism 5.01. Very occasionally, data were excluded from statistical analysis due to its extreme value.

## Electronic supplementary material

supplementary figures and tables
