# Supplementary material for: The Efficacy and Pharmacological Mechanism of Zn7MT3 to Protect against Alzheimer’s Disease
Source: Sci Rep. 2017 Oct 23;7:13763. doi: 10.1038/s41598-017-12800-x (PMC5653791; doi:10.1038/s41598-017-12800-x)
Supplement: Supplementary file 1 — supplementary figures and tables [file 41598_2017_12800_MOESM1_ESM.pdf]

The Efficacy and Pharmacological Mechanism of Zn_7MT3 to Protect against Alzheimer's Disease

Wei Xu, Qiming Xu, Hao Cheng and Xiangshi Tan

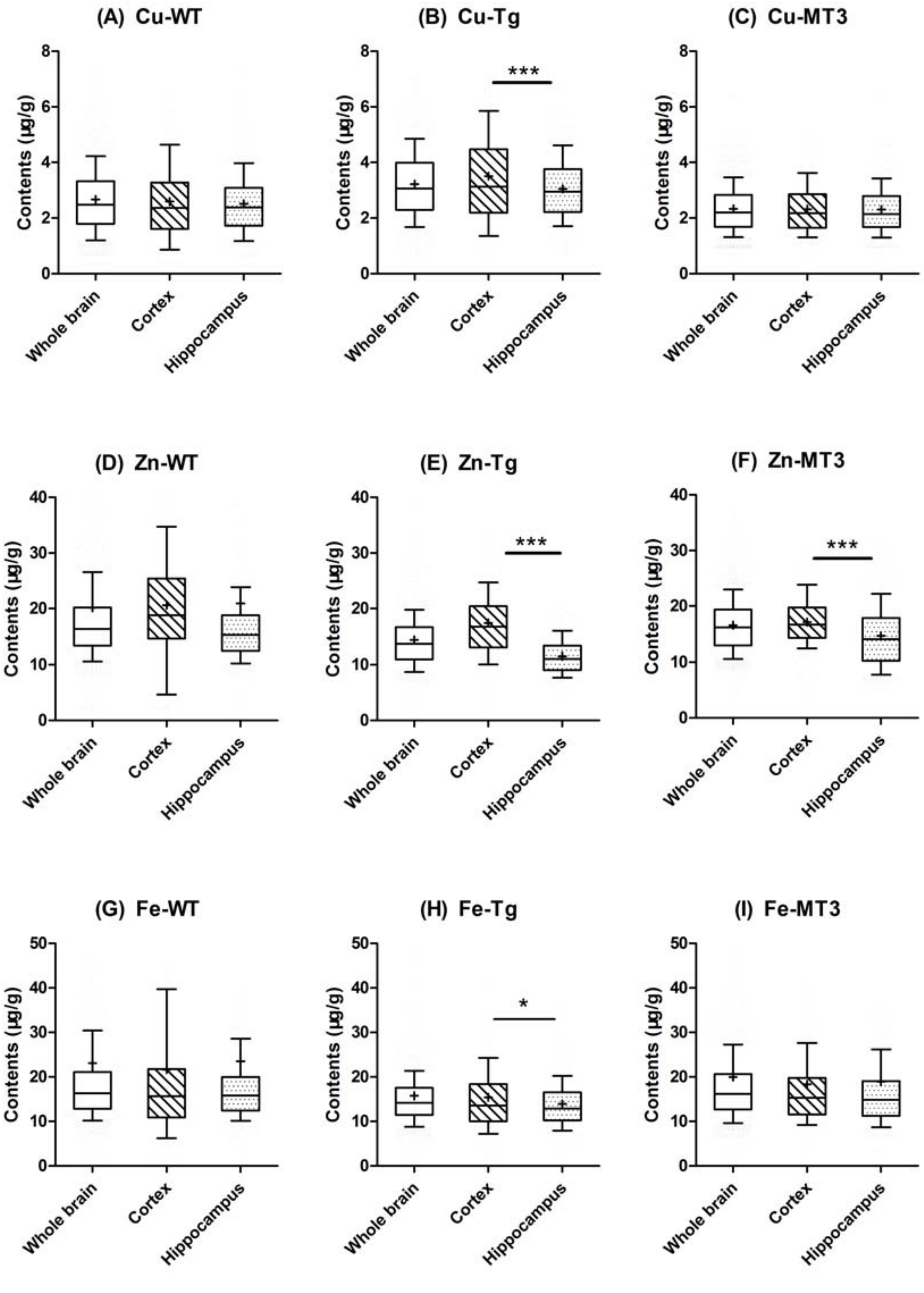

Figure S1. Quantitative analysis revealed metal distributions of Cu, Zn and Fe in whole brains, cortexes or hippocampi in different groups, respectively. Data were given as means ± SEM (n=1600-2000, 350-400 and 600-800 scanned spots for whole brain, cortex and hippocampus, respectively, $^{***}p < 0.001$, $^{*}p < 0.05$). Each group included 6 animals. Exact P values were shown in Table S8.

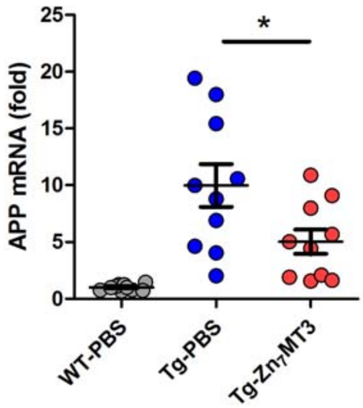

Figure S2. Chronic Zn_7MT3 treatment inhibits gene expression of APP. The expression level of APP was determined by RT-PCR. Tg-Zn_7MT3 mice showed notably lower APP expression level compared to Tg-PBS group. Data was given as means ± SEM (n=10 animals per group, *$p < 0.05$). Exact P values were shown in Table S9.

Table S1 Statistical analyses of Morris water maze test and step-down type passive avoidance test.

A. Statistical analyses of average escape latencies in the 6th day acquisition training.

WT-PBS vs. Tg-PBS, $p < 0.001$
Tg-PBS vs. Tg-Zn_7MT3, $p = 0.026$
Tg-Zn_7MT3 vs. WT-PBS, $p = 0.231$

B. Statistical analyses of crossing times in Morris water maze test.

WT-PBS vs. Tg-PBS, $p < 0.001$
Tg-PBS vs. Tg-Zn_7MT3, $p = 0.004$
Tg-Zn_7MT3 vs. WT-PBS, $p = 0.004$

C. Statistical analyses of time in target quadrant in Morris water maze test.

WT-PBS vs. Tg-PBS, $p < 0.001$
Tg-PBS vs. Tg-Zn_7MT3, $p = 0.041$
Tg-Zn_7MT3 vs. WT-PBS, $p = 0.153$

D. Statistical analyses of latency time in step-down type passive avoidance test.

WT-PBS vs. Tg-PBS, $p < 0.001$

Tg-PBS vs. Tg-Zn_7MT3, $p = 0.018$

Tg-Zn_7MT3 vs. WT-PBS, $p = 0.276$

E. Statistical analyses of error times in step-down type passive avoidance test.

WT-PBS vs. Tg-PBS, $p < 0.001$

Tg-PBS vs. Tg-Zn_7MT3, $p = 0.007$

Tg-Zn_7MT3 vs. WT-PBS, $p = 0.053$

Table S2 Statistical analyses in HE and Nissl staining

A. Statistical analyses of neuron cell area in HE staining.

WT-PBS vs. Tg-PBS, $p < 0.001$

Tg-PBS vs. Tg-Zn_7MT3, $p < 0.001$

Tg-Zn_7MT3 vs. WT-PBS, $p = 0.057$

B. Statistical analyses of Nissl positive cells in Nissl staining.

WT-PBS vs. Tg-PBS, $p < 0.001$

Tg-PBS vs. Tg-Zn_7MT3, $p < 0.001$

Tg-Zn_7MT3 vs. WT-PBS, $p = 0.120$

Table S3 Statistical analyses of TUNEL-positive cells in TUNEL staining.

WT-PBS vs. Tg-PBS, $p < 0.001$

Tg-PBS vs. Tg-Zn_7MT3, $p < 0.001$

Tg-Zn_7MT3 vs. WT-PBS, $p = 0.007$

Table S4 Statistical analyses of Aβ in brain tissues and plasma.

A. Statistical analyses of Aβ plaque number in ThS staining.

WT-PBS vs. Tg-PBS, $p < 0.001$

Tg-PBS vs. Tg-Zn_7MT3, $p = 0.012$

Tg-Zn_7MT3 vs. WT-PBS, $p < 0.001$

B. Statistical analyses of Aβ plaque area in ThS staining.

WT-PBS vs. Tg-PBS, $p < 0.001$

Tg-PBS vs. Tg-Zn_7MT3, $p = 0.010$

Tg-Zn_7MT3 vs. WT-PBS, $p < 0.001$

C. Statistical analyses of Aβ concentration in plasma.

WT-PBS vs. Tg-PBS, $p < 0.001$

Tg-PBS vs. Tg-Zn_7MT3, $p < 0.001$

Tg-Zn_7MT3 vs. WT-PBS, $p = 0.523$

Table S5 Statistical analyses of Cu, Zn and Fe concentration in brain tissues.

A. Statistical analyses of Cu in whole brain.

WT-PBS vs. Tg-PBS, $p < 0.001$

Tg-PBS vs. Tg-Zn_7MT3, $p < 0.001$

Tg-Zn_7MT3 vs. WT-PBS, $p < 0.001$

B. Statistical analyses of Cu in cortex.

WT-PBS vs. Tg-PBS, $p < 0.001$

Tg-PBS vs. Tg-Zn_7MT3, $p < 0.001$

Tg-Zn_7MT3 vs. WT-PBS, $p = 0.015$

C. Statistical analyses of Cu in hippocampus.

WT-PBS vs. Tg-PBS, $p < 0.001$

Tg-PBS vs. Tg-Zn_7MT3, $p < 0.001$

Tg-Zn_7MT3 vs. WT-PBS, $p < 0.001$

D. Statistical analyses of Zn in whole brain.

WT-PBS vs. Tg-PBS, $p < 0.001$

Tg-PBS vs. Tg-Zn_7MT3, $p < 0.001$

Tg-Zn_7MT3 vs. WT-PBS, $p < 0.001$

E. Statistical analyses of Zn in cortex.

WT-PBS vs. Tg-PBS, $p < 0.001$

Tg-PBS vs. Tg-Zn_7MT3, $p = 0.921$

Tg-Zn_7MT3 vs. WT-PBS, $p < 0.001$

F. Statistical analyses of Zn in hippocampus.

WT-PBS vs. Tg-PBS, $p < 0.001$

Tg-PBS vs. Tg-Zn_7MT3, $p < 0.001$

Tg-Zn_7MT3 vs. WT-PBS, $p < 0.001$

G. Statistical analyses of Fe in whole brain.

WT-PBS vs. Tg-PBS, $p < 0.001$

Tg-PBS vs. Tg-Zn_7MT3, $p < 0.001$

Tg-Zn_7MT3 vs. WT-PBS, $p = 0.006$

H. Statistical analyses of Fe in cortex.

WT-PBS vs. Tg-PBS, $p < 0.001$

Tg-PBS vs. Tg-Zn_7MT3, $p = 0.009$

Tg-Zn_7MT3 vs. WT-PBS, $p = 0.097$

I. Statistical analyses of Fe in hippocampus.

WT-PBS vs. Tg-PBS, $p < 0.001$

Tg-PBS vs. Tg-Zn_7MT3, $p < 0.001$

Tg-Zn_7MT3 vs. WT-PBS, $p = 0.039$

Table S6 Statistical analyses of oxidative stress level in brain.

A. Statistical analyses of MDA concentration.

WT-PBS vs. Tg-PBS, $p < 0.001$

Tg-PBS vs. Tg-Zn_7MT3, $p = 0.004$

Tg-Zn_7MT3 vs. WT-PBS, $p = 0.430$

B. Statistical analyses of 8-OHdG concentration.

WT-PBS vs. Tg-PBS, $p = 0.007$

Tg-PBS vs. Tg-Zn_7MT3, $p = 0.017$

Tg-Zn_7MT3 vs. WT-PBS, $p = 0.878$

Table S7 Statistical analyses of ^{15}N abundance.

WT vs. WT-(^{15}N-Zn_7MT3), $p = 1.000$

Tg vs. Tg-(^{15}N-Zn_7MT3), $p = 0.001$

Table S8 Statistical analyses of Cu, Zn and Fe distribution.

A. Statistical analyses of Cu distribution in WT-PBS group.

Whole brain vs. Cortex, $p = 0.767$

Cortex vs. Hippocampus, $p = 0.769$

Hippocampus vs. Whole brain , $p = 0.007$

B. Statistical analyses of Cu distribution in Tg-PBS group.

Whole brain vs. Cortex, $p = 0.057$

Cortex vs. Hippocampus, $p = 0.001$

Hippocampus vs. Whole brain , $p = 0.009$

C. Statistical analyses of Cu distribution in Tg-Zn_7MT3 group.

Whole brain vs. Cortex, $p = 0.983$

Cortex vs. Hippocampus, $p = 0.951$

Hippocampus vs. Whole brain , $p = 0.754$

D. Statistical analyses of Zn distribution in WT-PBS group.

Whole brain vs. Cortex, $p = 0.001$

Cortex vs. Hippocampus, $p = 0.980$

Hippocampus vs. Whole brain , $p = 0.055$

E. Statistical analyses of Zn distribution in Tg-PBS group.

Whole brain vs. Cortex, $p < 0.001$

Cortex vs. Hippocampus, $p < 0.001$

Hippocampus vs. Whole brain , $p < 0.001$

F. Statistical analyses of Zn distribution in Tg-Zn_7MT3 group.

Whole brain vs. Cortex, $p = 0.094$

Cortex vs. Hippocampus, $p < 0.001$

Hippocampus vs. Whole brain , $p < 0.001$

G. Statistical analyses of Fe distribution in WT-PBS group.
Whole brain vs. Cortex, $p = 0.746$
Cortex vs. Hippocampus, $p = 0.678$
Hippocampus vs. Whole brain , $p = 0.960$

H. Statistical analyses of Fe distribution in Tg-PBS group.
Whole brain vs. Cortex, $p = 0.915$
Cortex vs. Hippocampus, $p = 0.027$
Hippocampus vs. Whole brain , $p < 0.001$

I. Statistical analyses of Fe distribution in Tg-Zn_7MT3 group.
Whole brain vs. Cortex, $p = 0.550$
Cortex vs. Hippocampus, $p = 0.934$
Hippocampus vs. Whole brain , $p = 0.631$

Table S9 Statistical analyses of APP gene expression level.
WT-PBS vs. Tg-PBS, $p = 0.003$
Tg-PBS vs. Tg-Zn_7MT3, $p = 0.025$
Tg-Zn_7MT3 vs. WT-PBS, $p = 0.078$
